# The genomic case against genetic determinism

**DOI:** 10.1371/journal.pbio.3002510

**Published:** 2024-02-27

**Authors:** Gene E. Robinson, Rina Bliss, Matthew E. Hudson

**Affiliations:** 1 Carl R. Woese Institute for Genomic Biology, University of Illinois Urbana Champaign, Urbana, Illinois, United States of America; 2 Department of Entomology, University of Illinois Urbana Champaign, Urbana, Illinois, United States of America; 3 Neuroscience Program, University of Illinois Urbana Champaign, Urbana, Illinois, United States of America; 4 Department of Sociology, Rutgers University, New Brunswick, New Jersey, United States of America; 5 Department of Crop Sciences, University of Illinois Urbana Champaign, Urbana, Illinois, United States of America

## Abstract

This Perspective suggests that a deeper understanding of genotype by environment interactions at the molecular level could be a potent antidote to societal concerns of genetic determinism. Developing this idea for human social and behavioral genomics will require a paradigm that extends beyond traditional GWAS.

## Teaser

Animal studies reveal that the molecular wiring of the brain can be altered by heredity, the environment, and their interaction. A deeper molecular understanding of these interactions could be a potent antidote to societal concerns of genetic determinism for human behavior, but this requires a paradigm that extends beyond traditional genome-wide association study (GWAS).

Elucidating the relationship between genes and behavior is a grand challenge, with great opportunities for scientific and medical advances, and great risks for society. Several dark chapters of our history vividly illustrate the dangers of genetic determinism for behavior, which ascribes a dominant and oversimplified role to heredity. Genetic determinism contributed to the rise of eugenics, a term coined by the geneticist Francis Galton for the concept that selective breeding could improve the human species. Eugenics led to horrific effects on minority populations in the United States and other countries in the 19th and 20th centuries.

It is therefore a serious concern that a return to deterministic thinking has been used in the rationalization of heinous acts such as the racially motivated killings in Buffalo, New York in 2022. These misrepresentations also are starting to exert a chilling backlash effect, with objections to this type of research raised in scientific circles [1]. To help ensure a productive, sustainable, and socially acceptable research infrastructure, there is an urgent need for research paradigms that better integrate hereditary and environmental influences in the study of human behavior.

As discussed below, genes influencing behavior operate within gene regulatory networks that respond flexibly, contextually, and stochastically, not deterministically. However, most genome-wide association studies (GWAS) for behavior are not constructed to capture this dynamism. GWAS has been very successful when it is possible to precisely control the environment, especially in plant and animal sciences. GWAS in humans is especially challenging for the study of behavior because of the lack of control researchers have over both the population and its environment. Although behavioral geneticists are keenly aware of the importance of environmental influences on behavior, and generally include caveats against overly genetically deterministic interpretations of their work, these caveats are easily overlooked. The problem is that we do not yet have a comprehensive understanding of how environmental variation interacts with genetic variation at the molecular level to cause individual differences in behavior.

One approach to address this problem is to expand the use of environmental information when using GWAS. In fruit flies, GWAS analyses of aggression in different selected lines have revealed strong interactions between variation in genotype and variation in social environment [2]. Early efforts to move toward a Genome-wide by Environment Interaction Study framework in humans have involved coupling advanced analytics and creative methods to investigate environmentally plastic traits such as insomnia and body mass index. Geographic location and the Townsend deprivation index can be used as proxies for limited environmental parameters, and the CHARGE Gene-Lifestyle Interactions Working Group conducts various genome-wide interaction studies. This approach will become more powerful for human behavior as more metadata on non-genetic variables are collected for genetic repositories.

A second approach is to better integrate information from animal studies into the formulation of human studies. In animal studies, the ability to precisely manipulate the environment has revealed insights not possible to obtain with humans. Stochastic effects on the emergence of individual differences are evident in experiments performed with clonal Amazon molly fish [3]. In addition, the molecular wiring of the brain can be altered by both environment and genotype. Examples of the former include a report of effects of social dominance on brain gene expression in mice [4] and a study of carpenter ants showing that social network position influences brain gene expression and behavior [5]. In honey bees, colonies vary greatly in how aggressively they defend themselves from attack, and this trait has both inherited and environmental components. Individuals from aggressive genetic backgrounds reared by gentle colonies become more gentle, and vice versa. Moreover, individuals from aggressive and gentle colonies show differences in the structure of their brain gene regulatory networks [6]. As in ref. [7], different studies conducted for the same behavioral trait in different environments are likely to highlight different genes, with the impact of specific DNA variants more pronounced in some environments relative to others. This is consistent with recent empirical and conceptual analyses pointing to the problem of “missing regulation” [8], perhaps because gene regulatory networks for many different physiological and anatomical traits impact behavior indirectly [9]. Similarly, genetic variants significantly associated with educational attainment in some populations do not show the same associations in other populations. This has been attributed to differences in allele frequencies [10], but we think it is likely that genotype by environment interactions also play an important role. Many genetic differences do not directly control a trait, but instead control the way in which that trait responds to different environmental stimuli.

By no means are all social scientists, human geneticists, and animal biologists in sync with each other on this important topic. One sign of this disconnect is that different communities have used the term “sociogenomics” differently. Drawing on animal studies, this term was first introduced in 2005 by Robinson and colleagues [11], with an emphasis on environmental effects on genes and behavior. By contrast, Bliss’ survey of human social behavioral genomics a decade later, which did not refer to the animal literature [12], used the same term for human GWAS studies that ran the risk of genetic determinism. Given that animal and human social behavior share common neurobiological and molecular substrates [13], diverse, multidisciplinary scientific teams are needed to best exploit both the similarities and differences between humans and nonhuman animals at the behavioral, neurobiological, and molecular levels.

More speculatively, we suggest that emerging cellular and molecular technologies applied to human systems, both in vitro and in vivo, might be able to reveal mechanistic linkages between genetic and environmental variation. These include single-cell genomics, brain organoids, and new forms of non-invasive human brain imaging to capture molecular changes that occur as a result of differences in environmental exposure. It will be interesting to see whether any of these approaches can elucidate molecular mechanisms for environmental influences on human behavior, and thus further explicate the idea that genetic determinism is an overly simplistic explanation for human behavioral variation.

The field of human social and behavioral genomics needs to go beyond traditional GWAS to understand the complex relationship between genes and behavior. It should embrace Nobel Laureate Barbara McClintock’s vision of the genome “as a highly sensitive organ of the cell” [14], with even the most fundamental elements of gene regulation not fixed and subject to both environmental and genetic influence. The approaches highlighted here hold promise to contribute to a new synthesis that can address the problem of the causes and consequences of individual variation in human behavior. Because the political and humanitarian stakes are so high, scientists from different disciplines and with different perspectives must come together to expand the scope of their studies.
